# Anti-Neurotrophic Effects from Autoantibodies in Adult Diabetes Having Primary Open Angle Glaucoma or Dementia

**DOI:** 10.3389/fendo.2013.00058

**Published:** 2013-05-15

**Authors:** Mark B. Zimering, Thomas E. Moritz, Robert J. Donnelly

**Affiliations:** ^1^Medical Service, New Jersey Health Care System, Department of Veterans AffairsLyons, NJ, USA; ^2^Robert Wood Johnson Medical School, University of Medicine and Dentistry of New JerseyNew Brunswick, NJ, USA; ^3^Cooperative Study Coordinating Center, Hines Veterans HospitalHines, IL, USA; ^4^Molecular Resource Facility, University of Medicine and Dentistry of New Jersey, New Jersey Medical SchoolNewark, NJ, USA

**Keywords:** autoantibodies, diabetes mellitus, open angle glaucoma, dementia, neurite outgrowth

## Abstract

**Aim:** To test for anti-endothelial and anti-neurotrophic effects from autoantibodies in subsets of diabetes having open-angle glaucoma, dementia, or control subjects.

**Methods:** Protein-A eluates from plasma of 20 diabetic subjects having glaucoma or suspects and 34 age-matched controls were tested for effects on neurite outgrowth in rat pheochromocytoma PC12 cells or endothelial cell survival. The mechanism of the diabetic glaucoma autoantibodies’ neurite-inhibitory effect was investigated in co-incubations with the selective Rho kinase inhibitor Y27632 or the sulfated proteoglycan synthesis inhibitor sodium chlorate. Stored protein-A eluates from certain diabetic glaucoma or dementia subjects which contained long-lasting, highly stable cell inhibitory substances were characterized using mass spectrometry and amino acid sequencing.

**Results:** Diabetic primary open angle glaucoma (POAG) or suspects (*n* = 20) or diabetic dementia (*n* = 3) autoantibodies caused significantly greater mean inhibition of neurite outgrowth in PC12 cells (*p* < 0.0001) compared to autoantibodies in control diabetic (*n* = 24) or non-diabetic (*n* = 10) subjects without glaucoma (*p* < 0.01). Neurite inhibition by the diabetic glaucoma autoantibodies was completely abolished by 10 μM concentrations of Y27632 (*n* = 4). It was substantially reduced by 30 mM concentrations of sodium chlorate (*n* = 4). Peak, long-lasting activity survived storage ×5 years at 0–4°C and was associated with a restricted subtype of Ig kappa light chain. Diabetic glaucoma or dementia autoantibodies (*n* = 5) caused contraction and process retraction in quiescent cerebral cortical astrocytes effects which were blocked by 5 μM concentrations of Y27632.

**Conclusion:** These data suggest that autoantibodies in subsets of adult diabetes having POAG (glaucoma suspects) and/or dementia inhibit neurite outgrowth and promote a reactive astrocyte morphology by a mechanism which may involve activation of the RhoA/p160 ROCK signaling pathway.

## Introduction

Glaucoma is a chronic neurodegenerative disorder affecting retinal ganglion cells (RGC) and is a leading cause of blindness worldwide (Quigley, 2011). Glaucoma has been reported to increase in older adults with type 2 diabetes mellitus (Klein et al., 1994; Goldacre et al., 2012), and in certain populations having Alzheimer’s dementia (Tamura et al., 2006), although the mechanisms for these associations are unclear. The aim of the present study was to test the hypothesis that autoantibodies having anti-endothelial and anti-neuronal effects increase in older adults with type 2 diabetes and primary open angle glaucoma (POAG). We examined 20 older adults with type 2 diabetes and glaucoma, 34 age-matched adults without glaucoma (24 diabetic and 10 non-diabetic subjects), and 5 adults having diabetes and dementia for plasma autoantibodies which could inhibit neurite outgrowth in PC12 cells or decrease endothelial cell (EC) survival.

Humoral autoimmunity has been implicated in the etiology of certain forms of glaucoma (Tezel and Wax, 2004) including evidence for increased circulating autoantibodies to optic nerve head heparan sulfate proteoglycans (HSPG) in subsets of normal tension or POAG (Tezel et al., 1999). Anti-neuronal HSPG autoantibodies (having anti-neuronal and anti-endothelial effects) were reported in adult type 2 diabetes in association with certain microvascular complications including painful neuropathy (Zimering et al., 2011). The diabetic neuropathy plasma IgG autoantibodies induced EC contraction, EC apoptosis, and inhibited neurite outgrowth through a mechanism involving activation of the Rho A/Rho kinase signaling pathway (Zimering and Pan, 2009; Zimering et al., 2011). The Rho A/Rho kinase signaling pathway is present in diverse cell types (neurons, astrocytes, and endothelial-like trabecular meshwork cells) implicated in the pathophysiology of glaucoma (Tura et al., 2009; Kumar and Epstein, 2011; Kameda et al., 2012). Our hypothesis is that autoantibodies capable of binding to HSPG expressed on neurons, astrocytes, and ECs and activating Rho A/Rho kinase signaling may be capable of mediating axonal injury, ischemia, and glial reactivity underlying early glaucomatous changes (Crish and Calkins, 2011) leading to vision loss.

We now report that plasma IgG autoantibodies from diabetic POAG or suspects (*n* = 20) significantly inhibited neurite outgrowth in PC12 cells (*p* < 0.0001) compared to autoantibodies from diabetic (*n* = 24) or non-diabetic (*n* = 10) subjects without glaucoma. The neurite outgrowth inhibitory activity in four of four diabetic (POA) glaucomatous plasma autoantibodies tested was completely abolished by co-incubating PC12 cells with 10 μM concentrations of Y27632, a selective Rho kinase inhibitor. The neurite-inhibitory activity was also significantly reduced in the presence of 30 mM sodium chlorate which substantially reduces HSPG expression in PC12 cells. Taken together, these data suggest possible involvement of cell surface HSPG and activation of the Rho A/Rho kinase signaling pathway in the mechanism for PC12 neurite inhibition by diabetic glaucoma plasma autoantibodies.

## Materials and Methods

### Subjects

Informed consent for the Investigational Review Board (IRB) approved substudy to the Veterans Affairs Diabetes Trial (VADT) was obtained from all subjects. Fifteen of 89 subjects enrolled at the VA New Jersey Healthcare System (VANJHCS) study site to the VADT had a diagnosis of POAG or glaucoma suspect. Eleven of the 15 VADT subjects were included in the study based on the availability of an aliquot of plasma stored at −70°C necessary for protein-A affinity chromatography to obtain IgG autoantibodies. The four excluded subjects included two non-Hispanic white glaucoma subjects, one African-American glaucoma suspect and one non-Hispanic white glaucoma suspect. A control group of 21 VADT subjects without glaucoma or suspicion of glaucoma was randomly selected from among the remaining patients in whom baseline plasma was available for analysis. Twenty-two additional diabetic or non-diabetic glaucoma subjects or controls all evaluated in an IRB-approved VANJHCS study were selected for further analysis of the association between diabetic POAG and plasma autoantibodies.

### Diagnostic methods and subgroups

#### Glaucoma or suspects

All subjects were evaluated by the optometry and/or ophthalmology staff at the Veterans Affairs New Jersey Healthcare System. The diagnosis of POAG was based on findings from a combination of test modalities including: dilated fundoscopic examination, applanation tonometry, gonioscopy, pachymetry, periodic Humphrey visual field testing, or the results of Humphrey visual field tests, dilated fundoscopic examination, and tonometry performed by outside ophthalmologists and reported to optometry/ophthalmology specialists at the VANJHCS. Glaucoma suspect is defined as a high risk individual with asymmetric cup to disk (C/D) enlargement, equivocal neural rim narrowing, or retinal nerve fiber layer thinning, but without definite evidence for visual field loss. Subjects with increased intraocular pressure (IOP) alone, but without C/D enlargement were not included in the primary analysis, e.g., ocular hypertension (*n* = 1). Because many patients were already being treated with IOP-lowering medications at the time of study enrollment, it was not possible to distinguish subgroups of normal tension glaucoma vs. high-pressure POAG. Patients with secondary forms of glaucoma such as neovascular glaucoma (*n* = 1), or corticosteroid-associated glaucoma (*n* = 1) were excluded from the analysis. Subjects with other ocular or neuropathologies previously associated with potent EC autoantibodies, but lacking diabetic painful neuropathy, e.g., central retinal artery occlusion (*n* = 1), dementia (*n* = 1), or stroke (*n* = 1) were excluded from the analysis. Painful diabetic neuropathy was defined as the presence of characteristic findings on clinical examinations performed by expert neurologists as previously reported (Zimering et al., 2011). Diabetic nephropathy was defined as urinary albumin excretion ≥300 mg/g creatinine or urinary protein excretion ≥500 mg/g creatinine.

#### Diabetes and dementia (*n* = 3 + 2)

**Figure 1:**
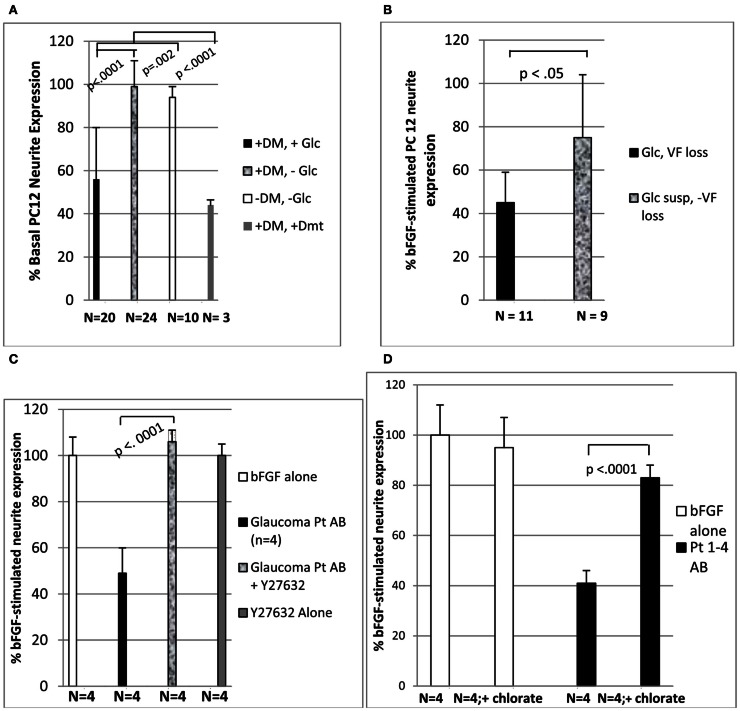
**Diabetic glaucomatous autoantibodies inhibit neurite expression in PC 12 cells (A) highest neurite-inhibitory activity was associated with glaucomatous vision loss (B) and was eliminated by treatment with Y27632 (C) or 30 mM sodium chlorate (D)**. **(A-D)** Thirty microgram per milliliter concentrations of the protein-A eluate fraction of plasma was incubated with PC12 cells in the presence or absence of 10 μM concentrations of Y27632 **(C)** or 30 mM concentrations of sodium chlorate **(D)** as described in Section “Materials and Methods.” Results (mean ± 1 SD of four different protein-A eluates) are expressed as % neurite expression compared to cells incubated with 10 ng/mL concentrations of basic fibroblast growth factor alone [i.e., 100%, open bars **(C,D)**]. DM, diabetes mellitus; Glc, glaucoma or suspect (susp); Dmt, dementia; VF, visual field.

In three VADT patients who developed dementia at the end of the clinical trial, neurite-inhibitory activity in PC12 cells was analyzed (Figure 1) as a control for the results in glaucoma, another type of chronic neurodegenerative disorder. Patient #1 was a 68-year-old African-American male with POAG, long-standing type 2 diabetes without significant diabetic retinopathy who was diagnosed with glaucoma at the onset of his VADT participation. His mild visual field loss was stable over a 7-year observation period; he was diagnosed with Alzheimer’s type dementia. Patient #2 is a 71 year old non-Hispanic white male with type 2 diabetes, ocular hypertension without baseline visual field loss whose visual fields were stable and cup/disk ratio was normal over a 5-year observation period; he was diagnosed with dementia secondary to traumatic brain injury. Patient #3 was a 75-year-old non-Hispanic white male with long-standing type 2 diabetes, no evidence of glaucoma, and mixed fronto-temporal and multi-infarct dementia.

Two additional subjects having diabetes and dementia were included in the analysis of EC inhibitory activity in stored protein-A eluates (Table 3). Patient #4 is a 70-year-old VADT subject who had type 2 DM, mild diabetic retinopathy, and later developed Alzheimer’s type dementia. Patient #5 is a 53-year-old type 1 DM (non-VADT) patient who had a wide spectrum of microvascular complications including nephropathy leading to end stage renal disease, multiple recurrent small vessel strokes (Zimering, 2010) progressing to dementia, proliferative diabetic retinopathy, autonomic and painful neuropathy, and glaucoma possibly due to steroid use.

#### Blood drawing

Baseline plasma samples were obtained from study subjects prior to initiation of treatment in the VADT substudy. Plasma bFGF was determined with a sensitive specific two-site IRMA as previously described (Zimering et al., 2009a). Bioactivity in protein-A eluate fractions was previously shown to be stable for 5 years or longer at −20°C (Zimering and Thakker-Varia, 2002).

#### Neurite outgrowth inhibition assay

Undifferentiated rat pheochromocytoma PC12 cells obtained from ATCC (Manassa, VA, USA) were grown in DMEM containing 10% horse serum, 5% fetal calf serum, and 10 ng/mL bFGF was added in order to induce neuronal differentiation. Test protein-A eluate fractions (1:50 dilution = 30 μg/mL protein) were added to cells in duplicate or triplicate and incubated at 37°C for 48 h. Next the proportion of cells expressing a neurite of ≥2 cell diameters in length was counted and compared to the proportion of bFGF-stimulated neurite cell expression in dishes containing bFGF without added test fractions as previously described (Zimering et al., 2011). Rho kinase-dependence of the neurite inhibition was assessed by co-incubating cells in the presence or absence of 10 μM concentrations of Y27632, a selective inhibitor for the Rho-associated protein kinase, p160ROCK (Uehata et al., 1997). Dependence of neurite-inhibitory effects in the diabetic protein-A eluates on cell surface sulfated proteoglycans was tested by comparing results in to pheochromocytoma PC12 cells grown in the presence or absence of 30 mM sodium chlorate, a treatment which substantially reduces HSPG expression in the cells (Hoogewerf et al., 1991).

#### Endothelial cell survival assay

Bovine pulmonary artery ECs (Clonetics, Inc., San Diego, CA, USA) were grown in Medium 199 plus 10% fetal calf serum and EC growth medium (EGM, Clonetics, Inc., San Diego, CA, USA). EC survival in the presence or absence of diabetic plasma protein-A eluates (30 μg/mL) was assessed after 48 h incubation as previously described (Zimering et al., 2011). Results represent the mean (±1 SD) of quadruplicate determinations.

#### Human lymphoblast cell culture

Human lymphoblasts (GM 01500) from a patient having light chain amyloidosis (AL disease) were obtained from the Human Mutant Repository, Coriell Institute (Camden, NJ, USA), and grown in RPMI 1640 containing 2 mM l-glutamine, 10% fetal calf serum. The cell supernatant was subjected to sequential protein-A affinity chromatography followed by antihuman IgG affinity chromatography in order to obtain partially purified monoclonal human IgG for testing of EC bioactivity.

#### Protein-A affinity chromatography

The IgG fraction of plasma was isolated using protein-A affinity chromatography as previously reported (Zimering et al., 2011).

#### Antihuman IgG affinity chromatography

The protein-A eluate fraction obtained after applying 1.0 mL of the human lymphoblast (GM 01500) cell supernatant to a protein-A affinity column (1.0 mL) was adjusted to pH 7.0 by adding 1 M Tris pH 9.0. The eluate was then diluted 1:1 with an equal volume of 100 mmol/L Tris pH 8.0 and applied to a goat antihuman IgG agarose column (1.0 mL), washed with 5 mL of 100 mmol/L Tris pH 8.0, and eluted stepwise with 5× 1.0 mL aliquots of 100 mmol/L sodium citrate, pH 3.0. The second eluate fraction contained nearly all of the recovered protein and it was adjusted to pH 7.0 with 1 M Tris pH 9.0 prior to sterile filtration and testing of biological activity.

#### Cerebral cortical astrocytes

Rat cerebral cortices were dissociated and placed in polylysine-coated T-75 flasks containing minimal essential medium without phenol red and 15% fetal bovine serum (having low estradiol concentration)-NM-15. They were fed on day 3 with fresh NM-15 medium. On day 10, the flasks were shaken overnight at 250 rpm. The next day, the medium was removed, the cells were rinsed with warm PBS, and then fresh NM-15 containing 10 mM AraC was added to the cells. Two days later, the cells were trypsinized and plated at 250,000 cells per dish in polylysine-coated 35 mm dishes containing NM-15. After 2–3 weeks in serum-containing medium, a small percentage of the astrocytes (1–5%) spontaneously differentiated from a flat polygonal shape into cells having a “stellate” appearance, bearing one or more thick processes located proximal to the cell body or several thin long branching processes located distal to the cell body. The acute effects of diabetic plasma autoantibodies on astrocyte morphology were assessed in cells that had been maintained for 2–3 weeks in culture. Cell viability was good under these long-term culture conditions.

#### Time-lapse photomicroscopy

Stellate-appearing astrocytes (and surrounding less differentiated cells) were imaged under high power magnification using a Nikon TMS microscope at 200× magnification. Following bath application of a 1:100 dilution (3–20 μg/mL) of protein-A eluates from diabetic glaucoma or control subjects, cells were observed continuously for acute change in morphology. Baseline and follow-up images were captured using a Nikon camera connected to the microscope with a phototube every 5 min for a period up to 30–45 min after addition of protein-A eluates.

#### Heparin Sepharose affinity chromatography

Heparin Sepharose (HS) affinity chromatography was carried out as previously reported (Zimering et al., 2009a).

#### SELDI mass spectrometry

Mass spectrometry was carried out as previously reported (Zimering et al., 2011).

#### Protein sequencing

Amino acid sequencing was carried out on the Applied Biosystems (Foster City, CA, USA) Procise® 494 protein sequencer using standard Edman sequencing. The resulting chromatographs were analyzed using Model 610A software.

#### Furin digests of diabetic protein-A eluates

Two microliters of an aqueous solution containing human recombinant furin (≥2000 units/mL) was added to 40 μL of protein-A eluates (8 μg protein) from each of three diabetic subjects in buffer containing 100 mmol/L Tris, pH 7.0, and 200 μM calcium. Following 180 min incubation at 25°C, 4 μL aliquots of furin-treated or -untreated protein-A eluates were added in quadruplicate to ECs for testing of biological activity. The remaining aliquot of furin-treated and -untreated paired samples was subjected to mass spectrometry which revealed a decrease in the peak 23 kDa apparent light chain MW species (present in untreated samples) in paired samples subjected to furin digestion.

#### Chemicals

Protein-A agarose was obtained from Pierce Chemical Co. (Rockford, IL, USA). Goat antihuman IgG (whole molecule) agarose was obtained from Sigma Chemical Co. (St. Louis, MO, USA). Human recombinant furin was obtained from Sigma Chemical Co. (St. Louis, MO, USA). All other chemicals and reagents were analytical grade.

#### Protein determinations

Protein concentrations were determined by a bicinchoninic acid protein assay kit (Pierce Chemical Co., Rockford, IL, USA).

#### Statistics

All data are the mean ± 1 SD as indicated. Comparisons were made by paired and unpaired Student’s *t*-tests, or Fischer’s exact test as indicated.

## Results

### Association between diabetic glaucoma or glaucoma suspect and race

Diabetic subjects with POAG or POAG suspects included a significantly higher proportion of African-American or subjects of Afro-Caribbean descent compared to diabetes without glaucoma (Table 1). Mean baseline diastolic blood pressure was significantly higher in diabetes having POAG or suspects than in diabetes without glaucoma (Table 1). It was also higher in a control group of non-diabetic subjects without glaucoma (Table 1). There was a trend of a significant association (*p* = 0.06) between low plasma basic fibroblast growth factor (0–3.4 pg/mL) and an increased proportion of diabetic subjects having POAG or suspects vs. no POAG or suspect (100 vs. 69%; Table 1).

**Table 1:** **Baseline clinical characteristics in the study subjects**.

Risk factor	Diabetes			No diabetes
	No Glc (*N* = 24)	Glc or susp (*N* = 20)	*P*-value*	No Glc (*N* = 10)
Age (years)	65.2 ± 8.3	69.6 ± 12.3	0.16	68.0 ± 16.7
Race (NHW/AA/H)	(22/1/1)	(9/10/1)	0.0006^∧^	(5/4/1)
BMI (kg/m^2^)	32.0 ± 5.1	32.0 ± 6.9	0.99	31.2 ± 7.7
DM duration (years)	11.0 ± 6.9	13.9 ± 10.0	0.28	NT
Hba_1_c (%)	8.3 ± 1.4	8.3 ± 1.5	0.95	NT
Syst bp (mm Hg)	129.9 ± 10.9	133 ± 13.6	0.48	124 ± 7.4
Diast bp (mm Hg)	67.6 ± 8.9	74.6 ± 9.1	0.01	76 ± 7.0^a^
Total Chol (mg/dL)	172 ± 14	176 ± 41	0.78	174 ± 51
Low bFGF (%)	69 (16)	100 (11)	0.06^^^	NT
Insulin use (%)	39	60	0.18^^^	NT

Results are mean ± 1 SD or proportion; (), number tested; Glc, glaucoma; susp, suspect; bp, blood pressure; Chol, cholesterol; Low bFGF, basic fibroblast growth factor = <4 pg/mL; ^^^Fischer’s exact test; *P-value for comparison of diabetics with or without glaucoma. NHW, non-Hispanic white, AA, African-American, H, Hispanic, ^a^*P* = 0.016 compared to diabetes without glaucoma.

### Association between diabetic glaucoma (suspect) and painful neuropathy or family history of dementia

Low plasma basic fibroblast growth factor was associated with a significantly increased occurrence of EC inhibitory autoantibodies in older adult type 2 diabetes from the VADT (Zimering et al., 2009a). Since baseline EC inhibitory autoantibodies predicted an increased risk for certain microvascular complications in the VADT substudy (Zimering et al., 2009b), we tested for associations between diabetic POAG and EC inhibitory autoantibodies or co-morbid diabetic microvascular complications. A significantly higher proportion of diabetic subjects with POAG or suspects (65 vs. 25%) had inhibitory EC autoantibodies compared to the proportion of diabetic subjects without glaucoma (*p* = 0.014; Table 2). Painful neuropathy was significantly more prevalent among diabetic POAG or suspects compared to diabetes without POAG (*p* = 0.014; Table 2). First-degree relatives (mother, father) of diabetic POAG or suspects were significantly more likely to have had Alzheimer’s dementia as a contributory cause of death compared to the parents of diabetic subjects without glaucoma (3/11 vs. 0/20; *p* = 0.04, Table 2).

**Table 2:** **Association between diabetic glaucoma or suspect and co-morbid microvascular complications or family history of dementia**.

Risk factor	Diabetes		
	No Glc (*N* = 24)	Glc or susp (*N* = 20)	*P*-value*
ME, AMD (%)	12.5	30	0.26
Nephropathy (%)	25	15	0.48
Painful neuropathy (%)	25	65	0.014
Inhibitory EC Act^c^ (%)	25	65	0.014
FH of dementia (%)	0 (20)	27 (11)	0.04

Results are proportion; (), number tested; Glc, glaucoma, susp, suspect, *Fischer’s exact test, comparison of diabetics with or without glaucoma; ME, macular edema, AMD, age-related macular degeneration, FH, family history in a 1° relative of Alzheimer’s dementia; ^c^significant inhibitory endothelial cell (EC) activity, defined as ≤90% of basal cell number as described in Section “Materials and Methods.”

**Table 3:** **Effect of long-term storage of protein-A eluates or furin digestion on EC activity**.

Diabetic subset	Endothelial cell bioactivity (%)		
	Before	After	*P*-value*
**LONG-TERM STORAGE (9–60 MONTHS)**			
No glaucoma or susp (*n* = 4)	107.3 ± 6.1	108 ± 6.3	0.87
Glaucoma suspect (*n* = 4)	98.7 ± 5.3	86.8 ± 20.2	0.20
Macular edema/nephr. (*n* = 6)	81.2 ± 5.8^†^	86.5 ± 14.8	0.47
Dementia (*n* = 5)	81.2 ± 3.3^†^	66.5 ± 4.5 (2)	0.01
**FURIN**			
Pt 1: glaucoma + Alz dementia (*n* = 1)	80 ± 8	65 ± 3	0.02
Pt 2: OHT + dementia (*n* = 1)	82 ± 11	87 ± 4	0.63

Results are mean, ±1 SD, endothelial cell bioactivity was determined as described in Section “Materials and Methods.” *P-value from Student’s *t*-test comparing results before and after storage or furin treatment ^†^*P* < 0.0001 compared to mean activity (before storage) in group of glaucoma suspects. OHT, ocular hypertension.

### Neurite outgrowth inhibitory activity in diabetic protein-A eluates: Association with glaucomatous vision loss or dementia

Mean PC12 neurite outgrowth inhibitory activity in the protein-A eluates (1:50 dilution; ∼30 μg/mL) of plasma from diabetic POAG or suspects significantly exceeded mean inhibitory activity from diabetes without glaucoma (55.7 ± 24 vs. 99.0 ± 12%; *p* < 0.0001; Figure 1A). It was also significantly more inhibitory (*p* = 0.002) compared to mean activity in protein-A eluates from subjects without diabetes and without glaucoma (94.2 ± 5%; Figure 1A). The protein-A eluates of three diabetic subjects who later developed dementia had the most potent mean neurite-inhibitory activity (44 ± 3%). It significantly exceeded mean inhibitory activity in IgG from diabetes without glaucoma (*p* < 0.0001, Figure 1A). Diabetic glaucomatous vision loss (*n* = 11 subjects) was associated with significantly more inhibitory mean PC 12 neurite activity compared to mean activity in the protein-A eluates from nine diabetic POAG suspects (45.5 ± 14 vs. 74.6 ± 31; *p* < 0.05; Figure 1B).

### Effect of Rho kinase inhibitor on neurite-inhibitory activity in diabetic plasma protein-A eluates

The Rho A/Rho kinase signaling pathway plays an important role in axonal pathfinding during development (Dickson, 2001). Y27632 is a selective inhibitor of Rho kinase (Ishizaki et al., 2000). Diabetic glaucomatous plasma autoantibodies (30 μg/mL) from four African-American patients having moderate or severe progressive glaucomatous visual loss caused significant inhibition of neurite outgrowth in PC 12 cells (Figure 1C). The mean inhibitory activity from the four protein-A eluates was significantly reduced (*p* < 0.001) by co-incubating PC12 cells with 10 μM concentrations of Y27632 (Figure 1C). Similar concentrations of Y27632 alone had no significant effect on PC12 cell neurite expression compared to cells exposed to bFGF (10 ng/mL) without Y27632 (Figure 1C).

### Effect of reduced sulfated proteoglycan expression in PC 12 cells

Sodium chlorate substantially reduces the sulfation of proteoglycans in various cells (Hoogewerf et al., 1991). To test for possible involvement of sulfated proteoglycan (e.g., HSPG) in the mechanism for neurite inhibition in diabetic glaucomatous plasma protein-A eluates, PC12 cell cultured in the presence or absence of 30 mM sodium chlorate were incubated with the protein-A eluate fractions (30 μg/mL) of plasma from four diabetic subjects with POAG. The mean neurite-inhibitory activity in all four protein-A eluates (Pt 1–4 AB) (assayed individually) was significantly reduced (*P* < 0.0001) in the PC12 cells cultured in the presence of 30 mM sodium chlorate compared to PC12 cells grown in standard medium (DMEM with 1% FCS) in the absence of chlorate ion (solid bars, Figure 1D). PC12 cell viability was unaffected by the presence of 30 mM chlorate. Neurite expression did not differ significantly in cells grown in the presence of bFGF with or without 30 mM chlorate (open bars, Figure 1D).

### Effect of diabetic plasma (POA) glaucomatous autoantibodies on astrocyte morphology

**Figure 2:**
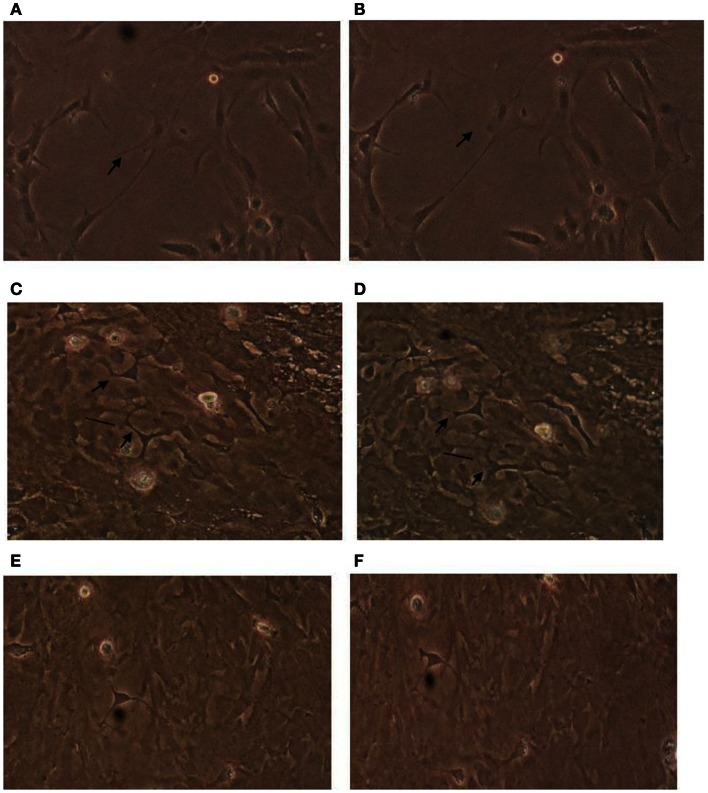
**Effect of diabetic glaucomatous autoantibodies on astrocyte morphology**. Morphology in cerebral cortical astrocytes **(A)** before or **(B)** 30 min after addition of 10 μg/mL concentration of protein-A eluate from type 2 DM and glaucoma; **(C)** before or **(D)** 10 min after addition of 3 μg/mL concentration of the protein-A eluate fraction from type 2 DM having glaucoma and Alzheimer’s type dementia (Pt 1). Antibodies caused varying degrees of withdrawal of thick astrocyte processes associated with increased stress fiber expression (arrows), and cell contraction (vertical lines). Astrocytes cultured in the presence of 10 μM concentrations of Y27632 for 10 min before **(E)** and after **(F)** addition of a 3 μg/mL concentration of the Pt 1 protein-A eluate fraction did not undergo similar changes in their appearance. Results similar to those shown in **(A–D)** were obtained in eight experiments using 3–10 μg/mL concentrations of autoantibodies from five different diabetic glaucoma patients. Much less if any acute change in morphology was observed (*n* = 4 experiments) using 10–20 μg/mL concentrations of protein-A eluate from four type 2 DM subjects without glaucoma.

Astrocytes play critical role(s) in maintenance of the blood-brain barrier (Allen et al., 2010), protect neurons against glutamate-induced excitotoxicity, and modulate blood flow to actively firing neurons. To facilitate these important neuronal support roles, astrocyte processes lie in close apposition to basement membranes, capillaries, and neuronal synapses. Since the expression of astrocytic processes, or stellation, is inhibited by activity in the RhoA/Rho kinase signaling pathway (Abe and Misawa, 2003), we next tested whether autoantibodies from diabetic POAG or control subjects could inhibit astrocyte stellation *in vitro*. Plasma autoantibodies (1:100 dilution = 3–20 μg/mL) from five of five diabetic glaucoma or suspects tested (*n* = 8 experiments) caused retraction of thick processes located proximal to the astrocyte cell body (e.g., Figures 2A,B). Withdrawal of proximally located processes was most rapid (occurring within 2–5 min) and more extensive leading to disruption connectivity among processes in neighboring cells after bath application of low concentration (3 μg/mL) of diabetic POAG plus dementia autoantibodies (Figures 2C,D). The retraction of thick astrocytic processes (induced by diabetic glaucoma + dementia autoantibodies) was prevented by pretreating cells (for 10 min) with 5 μM concentrations of Y27632 (Figures 2E,F; *n* = 2 experiments). Y27632 alone had no effect on astrocyte morphology (not shown in Figure 2). Control plasma autoantibodies (10–20 μg/mL) from four of four age-matched, non-diabetic subjects without glaucoma (or suspicion of glaucoma) tested had little or no significant effect on the morphology of astrocyte thick processes (*n* = 4 experiments, not shown in Figure 2). The active autoantibodies (10–20 μg/mL) from two of the diabetic glaucoma subjects that caused retraction of thick astrocyte processes had no apparent significant effect on morphology of thinner more distally located processes found in several highly differentiated, extensively branching astrocytes (*n* = 2 experiments, each observed for 30 min).

### Furin treatment unmasked potent endothelial cell inhibitory activity in diabetic dementia protein-A eluate

Mean EC inhibitory activity in the protein-A eluates from subsets of diabetic macular edema or nephropathy (*n* = 6) or in diabetic dementia plasma (*n* = 5) significantly exceeded mean activity in protein-A eluates from diabetic non-glaucoma or glaucoma suspects (Table 3). After 9 months storage of Patient 4 and 5 diabetic dementia protein-A eluates, a significantly more inhibitory form of EC activity appeared spontaneously (Table 3) having similar magnitude to the inhibitory activity recovered from the Patient 1 diabetic dementia and glaucoma protein-A eluate following brief exposure to the proprotein convertase (PC) furin (Table 3). Mass spectrometry or amino acid sequencing of long-term stored protein-A eluates from four of four diabetic dementia plasmas tested revealed either peak 23 kDa MW species or 86–89% amino acid sequence homology to human kappa (κ) light chain variable regions. In an adult type one DM patient (Patient 5) who suffered a severe unremitting course of microvascular and neuropathic complications (Zimering, 2010) including multi-infarct dementia and steroid-induced glaucoma, the protein-A eluate retained its potent neurite-inhibitory activity after 5 years storage at 0–4°C. Half-maximal inhibition in EC survival occurred at concentrations of Patient 5 IgG (30 nM) which were comparable to the half-maximal inhibitory concentrations (10 nM) observed in the purified monoclonal IgG_2_ kappa light chains obtained from a clone of human lymphoblasts (GM01500) from a patient having multiple myeloma and systemic light chain AL (data not shown). Using HS affinity chromatography, we were able to obtain a highly purified peak EC inhibitory fraction in the Patient 5 protein-A eluate which eluted from HS column with 0.1 M NaCL. Comparison of the amino acid sequences in the Patient 5 peak, HS-purified fraction to its starting material protein-A eluate or to AL disease monoclonal IgG_2_ kappa light chains revealed the presence of proline at amino acid position 12 in the peak HS-purified Patient 5 diabetic eluate only. Proline at amino acid position 12 is characteristic of a restricted subgroup of kappa II gene-encoded sequences (Wu and Kabat, 1970).

## Discussion

The present data are the first to suggest that plasma autoantibodies from a subset of older adult type 2 diabetes having POAG or suspects cause significant inhibition of neurite outgrowth in PC 12 cells via a mechanism which may involve binding to cell surface HSPG and activation of intracellular Rho kinase. Our results are consistent in part with earlier reports of increased titers of autoantibodies specific for (optic nerve head) HSPG in subsets of normal tension or primary angle glaucoma having a systemic autoimmune disease (Tezel et al., 1999).

Unlike systemic lupus erythematosus (SLE), type 2 diabetes is not a systemic autoimmune condition. Yet autoantibodies capable of inducing EC apoptosis were described in subsets of SLE nephropathy (van Paassen et al., 2007) or in older adult type 2 diabetes in association with macular edema, nephropathy, and/or painful neuropathy (Zimering and Pan, 2009). The present finding of a significant association between the occurrence of diabetic painful neuropathy and POAG is novel. Taken together with the chlorate sensitivity of the PC12 neurite-inhibitory activity in diabetic glaucoma autoantibodies it suggests HSPG (or a closely related sulfated proteoglycan) as a possible common target for autoantibodies in diabetic POAG and diabetic painful neuropathy.

Heparan sulfate glycosaminoglycans (GAGs) play important roles in early brain development (Inatani et al., 2003) including ensuring retinal axon guidance to the optic nerve head (Ogata-Iwao et al., 2011) and the correct topographic arrangement of axons in the optic nerve (Lee et al., 2004). HSPG are ubiquitously expressed on the cell surface of ECs, mesenchyme-derived cells and neurons. Following CNS injury, astrocyte HSPG increases its sulfation (Properzi et al., 2008). Basic fibroblast growth factor is a known neurotrophic and survival factor in RGC (Soto et al., 2006) which requires HSPG for its biological activity. Anti-HSPG autoantibodies may interfere with the survival-promoting effects of locally available bFGF in RGCs or contribute to generally low plasma bFGF in diabetic glaucoma. Autoantibody-induced neurite retraction may deprive optic axons of trophic support from postsynaptic target neurons. Reversal of astrocyte stellation in association with stress fiber expression and astrocyte contraction suggests that the diabetic glaucoma autoantibodies which induced morphological astrocyte reactivity (Tura et al., 2009) may lead to loss of glial-neuron interaction(s) which is an important source of trophic support in RGCs.

Ten of eleven diabetic subjects who suffered POAG-related visual field loss were African-American or of Afro-Caribbean origin, groups especially prone to develop POAG or glaucomatous blindness (Marshall, 1989). Our findings of generally low plasma bFGF among diabetic glaucoma or suspects is consistent with an earlier report of a significant association between low plasma bFGF and African-American race in a multi-ethnic VADT substudy of older adults having type 2 diabetes (Zimering et al., 2008). Higher mean diastolic blood pressure in the current diabetic glaucoma group may reflect (in part) inclusion of a higher proportion of African subjects. It is also consistent with a reported association between increased diastolic blood pressure and raised IOP in older adults having diabetes (Klein et al., 1994).

The prevalence of glaucoma among blacks living in certain geographically isolated areas is striking. It suggests possible involvement of population- or race-specific hereditary predisposition factor(s). For example, in the Barbados Eye study, the prevalence of POAG among older adult Afro-Caribbean men and women (age 70 years or above) was 17%; it was eight times higher than in Barbadian whites, and could not be accounted for by diabetes or hypertension (Leske et al., 1994). One possible contributory factor is known hereditable differences in autoantibody subclass concentrations. African-American children had higher concentrations of plasma total IgG and the IgG_2_ subclass compared to Caucasian children (Shackelford et al., 1985). Km1 is a marker in the constant region of kappa light chains which occurs with 3.7-fold higher frequency in African-American compared to Caucasian populations (Granoff et al., 1984). Km1 was associated with increased IgG_2_ antibody response to certain polysaccharide antigens in African-American children (Granoff et al., 1984); and the IgG_2_ antibody response (associated with Km1) was predominated by expression of a particular subgroup of less prevalent kappa light chain (κ IIa) genes (Lucas et al., 1991). A kappa II gene is predicted to have encoded the peak inhibitory kappa light chain in the eluate from (type 1 DM) Patient 5 having severe disease manifestations (Zimering, 2010). In contrast, in Caucasian adults, an increased IgG_2_ antibody response to several different common polysaccharide bacterial antigens was associated with Gm23, a marker in the heavy chain constant region (Ambrosino et al., 1985). Gm23 was reported to be associated with a significantly increased risk of diabetic retinopathy (Stewart et al., 1993). Our finding of a significantly increased risk for Alzheimer’s dementia among the parents of diabetic glaucoma or suspects is consistent with the possibility that a subset of autoantibodies having highly potent (neurite-, EC and glial-) inhibitory properties may reflect inheritance of a particular subgroup of kappa light chain genes. Increased prevalence of such genes as the kappa II (Ig) gene subgroup in persons of African descent may reflect an earlier evolutionary selection pressure that acted to increase the prevalence of a survival-promoting, beneficial allele. The later occurrence of slow neurodegenerative diseases such as open angle glaucoma or Alzheimer’s dementia in persons harboring potent kappa light chain autoantibodies is consistent with the concept of “antagonistic pleiotropy,” advanced by Williams (1957). Williams postulated that senescence-associated mortality occurs through the selection of genes which confer an early reproductive (survival) benefit yet harbor additional latent harmful effects to the organism which are expressed during aging.

Glaucoma is a slowly progressive neurodegenerative disease whose severity is significantly affected by elevated IOP (Sommer, 1996; Quigley, 2011). IOP is thought to mediate remodeling of the optic nerve head extracellular matrix (ECM) in part through the activation of membrane-type matrix metalloproteinases (MT-MMP) (Agapova et al., 2003). Furin is a proprotein convertase (PC) which is expressed in vascular endothelial and trabecular meshwork cells under conditions of increased hemodynamic or mechanical stress (Negishi et al., 2001; Remacle et al., 2008). In our prior work, long-term storage of certain cancer sera protein-A eluates was associated with the spontaneous appearance of highly potent EC inhibitory substances which were excitotoxic in rat embryonic hippocampal neurons and had MW and amino acid sequence characteristics of Ig kappa half-light chains (Zimering et al., 2011). The current preliminary data are consistent with the possibility that furin or a closely related PC which can recognize and cleave substrates at multi-basic amino acid sequences (Remacle et al., 2008) causes gain-in-inhibitory function through cleavage of certain IgGs at specific recognition sequences(s). One possible recognition site (perhaps giving rise to kappa half-light chains) is the K-(X)_3_-K-R sequence which is located in the variable-constant switch region in certain κ light chains. The beta site amyloid precursor protein cleaving enzyme 1 (Scholefield et al., 2003), BACE1 (β-secretase), has been implicated in Alzheimer’s disease pathogenesis because it generates amyloid β-peptide from its precursor protein. Of interest, β-secretase (BACE1) is under negative regulatory control through its binding to heparan sulfate (Scholefield et al., 2003). Certain members of the PC family are expressed at the cell surface in association with HSPG (Mayer et al., 2008). Thus localization of anti-HSPG autoantibodies at such cell regions might provide a mechanism for dual activation of β-secretase and for proteolytic processing of autoantibodies leading to more highly potent, long-lasting inhibitory substances.

A limitation of our study is that it is small and the results principally reflect the experience of older African-American men having POAG. More study in women and other racial groups is needed to confirm the present findings and to determine whether PC12 neurite-inhibitory autoantibodies may increase in non-diabetic glaucoma subjects. The mechanism(s) underlying glaucomatous neurodegeneration are complex and multifactorial (Kuehn et al., 2005). It is possible anti-HSPG autoantibodies may occur as a consequence of tissue injury yet have only a limited, bystander role in the neurodegenerative disease process. Although *in vivo* experimental support of a pathogenic role for anti-HSPG autoantibodies in glaucomatous neurodegeneration is lacking, *in vitro* effects of the autoantibodies in ECs, neurons and cerebral cortical astrocytes support a potential role in promoting neurodegeneration underlying glaucoma and dementia. Of interest, in a mouse model of hereditary glaucoma, high-dose irradiation unexpectedly completely prevented the subsequent development of glaucomatous RGC degeneration (Anderson et al., 2005). Basic FGF is released following irradiation and it protects microvascular endothelium against radiation-induced apoptosis (Fuks et al., 1994). More study in a mouse model of irradiation-induced neuroprotection could provide a test for involvement of bFGF or factors (anti-HSPG autoantibodies) which negatively affect bFGF local bioavailability in determining long-term RGC survival. Our data do not exclude the possible involvement of other kinds of autoreactive antibodies, such as heat shock proteins (HSP) autoantibodies, or cell-mediated immune mechanisms in retinal ganglion degeneration (Wax et al., 2008). For example, immunization with HSP 27 or HSP60 in the Lewis rat induced a glaucoma-like pattern of RGC degeneration via activation of T cells which secreted Fas ligand (Wax et al., 2008). Still evidence suggests that early activation of RGC survival pathways (perhaps mediated in part by bFGF) may prevent or delay Fas ligand-induced RGC apoptosis (Kim and Park, 2005).

The retinal inner limiting membrane (ILM) contains HSPG; and the ILM undergoes thickening during aging (Candiello et al., 2010) and in diabetes. In a subset of diabetic glaucoma or suspects (3 of 20 subjects), protein-A eluates caused mild EC stimulation suggesting that diabetic glaucomatous autoantibodies are heterogeneous and may include immune complexes. Trapping of immune complexes in the retinal ILM might disrupt its barrier function or lead to complement activation. A possible underlying role for humoral autoimmunity in subsets of dementia is suggested by reports that autoantibodies cross-reactive with vascular HSPG increased in serum from older adults having senile, Alzheimer’s type dementia (Fillit et al., 1987). Brain tissue from Alzheimer’s-type dementia patients showed diffuse deposition of IgG, fibrinogen, and glial hyperreactivity indicative of loss of EC barrier integrity (Ryu and McLarnon, 2009). More study is needed to determine whether plasma diabetic autoantibody PC12 neurite-inhibitory activity may be a useful (early) marker for sustained activation of Rho kinase in diverse cell types including neurons, astrocytes, or ECs involved in mediating slowly, progressive glaucomatous visual field loss, or in a subset of diabetes having dementia.

## Conflict of Interest Statement

The authors declare that the research was conducted in the absence of any commercial or financial relationships that could be construed as a potential conflict of interest.
